# Anti-Biofilm Effects of *Torilis japonica* Ethanol Extracts against *Staphylococcus aureus*

**DOI:** 10.4014/jmb.2107.07053

**Published:** 2021-11-28

**Authors:** Geun-Seop Kim, Chae-Rin Park, Ji-Eun Kim, Hong-Kook Kim, Byeong-Soo Kim

**Affiliations:** 1 Department of Integrated Life Science and Technology, Kongju National University, Yesan-gun, Chungnam 32439, Republic of Korea; 2 Department of Companion and Laboratory Animal Science, Kongju National University, Yesan-gun, Chungnam 32439, Republic of Korea

**Keywords:** Methicillin-resistant *Staphylococcus aureus*, quorum sensing, biofilm degradation, hemolysis, *Torilis japonica* extract

## Abstract

The spread of antibiotic-resistant strains of *Staphylococcus aureus*, a gram-positive opportunistic pathogen, has increased due to the frequent use of antibiotics. Inhibition of the quorum-sensing systems of biofilm-producing strains using plant extracts represents an efficient approach for controlling infections. *Torilis japonica* is a medicinal herb showing various bioactivities; however, no studies have reported the anti-biofilm effects of *T. japonica* extracts against drug-resistant *S. aureus*. In this study, we evaluated the inhibitory effects of *T. japonica* ethanol extract (TJE) on biofilm production in methicillin-sensitive *S. aureus* (MSSA) KCTC 1927, methicillin-resistant *S. aureus* (MRSA) KCCM 40510, and MRSA KCCM 40511. Biofilm assays showed that TJE could inhibit biofilm formation in all strains. Furthermore, the hemolysis of sheep blood was found to be reduced when the strains were treated with TJE. The mRNA expression of *agrA*, *sarA*, *icaA*, *hla*, and *RNAIII* was evaluated using reverse transcription-polymerase chain reaction to determine the effect of TJE on the regulation of genes encoding quorum sensing-related virulence factors in MSSA and MRSA. The expression of *hla* reduced in a concentration-dependent manner upon treatment with TJE. Moreover, the expression levels of other genes were significantly reduced compared to those in the control group. In conclusion, TJE can suppress biofilm formation and virulence factor-related gene expression in MSSA and MRSA strains. The extract may therefore be used to develop treatments for infections caused by antibiotic-resistant *S. aureus*.

## Introduction

*Staphylococcus aureus* is a gram-positive bacterium present on the skin and in the digestive tract of humans, and acts as an opportunistic pathogen that causes various diseases such as food poisoning, atopic dermatitis, and pneumonia, which may be fatal in serious cases [1-3]. At present, the long-term use of antibiotics has resulted in the development of antibiotic-resistant *S. aureus* strains that are associated with various clinical issues such as treatment failure and transmission of antibiotics resistance [4, 5]. Furthermore, *S. aureus* forms biofilms which may confer antibiotic resistance, making infections with these strains even more difficult to treat [6].

A biofilm is a collection of bacterial polymeric compounds, typically consisting of proteins, extracellular DNA, and polysaccharides, which adhere together and to a specific surface [7]. Biofilm-forming bacteria show higher resistance to antibiotics than that of planktonic bacteria, resulting in chronic or re-infection [8]. Biofilms are produced when bacteria cannot withstand the external environment, such as in the presence of antibiotics or immune cells [9]. In infections with *S. aureus*, biofilms protect the bacteria from white blood cells and antibiotics [10]. This change in state is regulated by a special mechanism called quorum sensing (QS) [11]. QS involves cell-to-cell signaling, and *S. aureus* QS is controlled by the *agr* system, which directly or indirectly regulates the *S. aureus* biofilm-related metabolism and the generation of virulence factors, thereby contributing to infection related to biofilm formation [12, 13]. QS is a key process in the development of *S. aureus* infections, and the *agr* system is used as a modulator [14]. *agr* increases the production of many virulence factors and decreases the production of colonization factor, subsequently increasing the viability of *S. aureus* in poor environmental conditions [15, 16]. The *agr* operon is activated by SarA [17]. The phosphorylation of *agrA* induces the transcription of *RNAIII*, and the transcription of the two divergent genes *RNAII* and *RNAIII* is activated by the promoters P2 and P3, respectively [7, 18, 19]. Most virulence genes regulated by *RNAIII* are additionally regulated by SarA. *agrA* and *RNAIII* represent important QS effector genes [7]. *RNAIII* encodes *hla* in high-density bacterial populations and is also involved in the production of the α-hemolysin called δ toxin [20, 21]. In addition to *agr*, the gene *icaA* affects biofilm formation [22]; *ica* is involved in the accumulation stage of biofilm formation by inducing the biosynthesis of polysaccharide intercellular adhesin [23].

The pathogenicity of pathogens through biofilm and QS increases various virulence factors that threaten health [6, 8]. Many pathogens, including *S. aureus*, use QS to control toxicity and form biofilm, and therefore, the blocking of toxicity control mechanisms by natural products can be applied in various ways to control microbial infection [24]. Plants can control the growth and attack of pathogens by producing secondary metabolites, and unlike conventional antibiotics, these can be expected to exert control over the colonization of pathogens and the ability of pathogens to produce toxins [43]. Several studies have shown that *S. aureus*-derived toxins and toxic factors associated with QS can be controlled using a variety of natural product extracts [24, 25]. However, there are few studies evaluating the effect of *Torilis japonica* on QS in *S. aureus*. *T. japonica* is an herb that grows naturally in mountains and fields across Korea and includes a sequiterpene acetate compound, a hemiterhenoid compound and a flavonoid compound [27, 44, 45]. Because *T. japonica* contains bioactive compounds, it has various beneficial effects such as pain relief and also shows antioxidant, anti-inflammatory, anti-obesity, and anti-cancer activities [26-28]. *T. japonica* has also been reported as having anti-bacterial activities against gram-positive and gram-negative bacteria [32, 33]. Based on these bioactive properties of *T. japonica*, we investigated the effects of *T. japonica* ethanol extract (TJE) on biofilm formation and virulence factor control in *S. aureus*.

## Materials and Methods

### Bacterial Strains

Methicillin-sensitive *S. aureus* (MSSA) KCTC 1927, and methicillin-resistant *S. aureus* (MRSA) KCCM 40510 and KCCM 40511 were purchased from the Korean Collection for Type Cultures (KCTC) and Korean Culture Center of Microorganisms (KCCM). The bacteria were cultured in tryptic soy broth (TSB; Difco, USA) at 37°C for 24 h and stored at -70°C in 15% glycerol stocks.

### Preparation of TJE

*T. japonica* was purchased from a farm located in Yeongcheon-si, Gyeongsangbuk-do, Korea. Dry *T. japonica* fruit (100 g) and 95% ethanol (1 L) were added to a flask following extraction at room temperature (25–28°C) for 72 h. TJE was filtered using filter paper and concentrated using a centrifugal vacuum concentrator (Hanil Scientific Inc., Korea). Dimethyl sulphoxide was used as a solvent.

### Bacteria Growth Assay

The bacteria viability assays for TJE against MSSA KCTC 1927, MRSA KCCM 40510, and MRSA KCCM 40511 were performed according to the dilution method [29]. *S. aureus* cultures were pre-incubated at 37°C in TSB, and the bacterial suspension was adjusted to the McFarland standard 0.5 using 1× phosphate-buffered saline (PBS; Gibco, USA). The prepared bacterial suspension was diluted 10-fold using Mueller-Hinton broth (Difco, USA) to prepare a final bacterial suspension. TJE was added to 96-well plates for each concentration, and the prepared bacterial suspensions were inoculated into each well. The 96-well plates were incubated at 37°C for 18 h, and optical density at 600 nm was measured using a spectrophotometer (Biotek, Korea) to calculate the MIC.

### Biofilm Assay

Biofilm formation assays were performed according to the crystal violet-based method [30]. *S. aureus* were pre-cultured in TSB for 18 h at 37°C, and the bacterial suspension was adjusted to the McFarland standard 0.5 using 1× PBS (Gibco). The cultures were incubated in 2% glucose TSB with or without TJE 37°C for 18 h, and then washed with tap water thrice. The biofilm was stained using 1% crystal violet dye for 20 min and washed with tap water thrice. The stained crystal violet was dissolved using 95% ethanol, and the optical density at 600 nm was measured using a spectrophotometer (Biotek, Korea). The biofilm inhibition rate (%) was calculated using the following equation:

Biofilm inhibition rate (%) = (1−OD600nm_sample_/OD600nm_control_) × 100

### Hemolysis Assay

Hemolysis assay was performed according to the sheep blood method described by Larzabal *et al*. [31] with certain modifications. Sheep red blood cells (RBCs) (Innovative Research, USA) were added to 1× Hanks' Balanced Salt Solution (Gibco) and centrifuged at 2,000 ×*g* for 3 min; this step was repeated thrice. All *S. aureus* cultures were incubated in TSB with or without samples for 12 h at 37°C. Culture supernatant (300 μl) was added to the RBC suspension (5 ml) and incubated at 37°C for 1 h. Cultured RBC suspensions were centrifuged at 16,600 ×*g* for 10 min, and then the optical density (543 nm) of the supernatant was measured using a spectrophotometer (Biotek). Hemolysis rate (%) was calculated using the following equation:

Hemolysis rate (%) = (1−OD543nm_sample_/OD543nm_control_) × 100

### Bacterial RNA Isolation and Reverse Transcription-Polymerase Chain Reaction (RT-PCR) Analysis

The effects of TJE on the regulation of QS-related genes (*agrA*, *sarA*, *icaA*, *hla*, *RNAIII*, and 16S rRNA) in MSSA KCTC 1927, MRSA KCCM 40510, and MRSA KCCM 40511 were determined via RT-PCR. All *S. aureus* cultures were pre-cultured in TSB medium at 37°C for 12 h, and then subjected to secondary culturing in the presence of TJE for 12 h. The culture medium (1 ml) was dispensed into 1.8 ml Eppendorf tubes and washed with PBS thrice at 12,000 ×*g* for 3 min. Subsequently, 5 μl of lysostaphin (1 mg/ml; Sigma-Aldrich, USA) was added to 195 μl of Tris-EDTA buffer (pH 8.0; Ambion, USA) and reacted at 37°C for 30 min. After the reaction, 1 ml of Trizol (Sigma-Aldrich, USA) and glass beads (approximately 212–300 μm; Sigma-Aldrich, USA) were added to the tube and vortexed for 30 min. The concentration of extracted total RNA was quantitated using a spectrophotometer (Biotek, Korea), and 1 μg of RNA was used to synthesize cDNA with a cDNA synthesis kit (Thermo Fisher, USA) according to the manufacturer’s protocol. PCR was performed as follows using the primers shown in Table 1: initial denaturation at 95°C for 5 min followed by 95°C for 25 s, annealing at primer specific temperature for 20 s, extension at 72°C for 30 s (28 cycles) and final extension at 72°C for 3 min. The PCR products were verified via electrophoresis for 20 min at 100V in 1.2% agarose gel. PCR bands were visualized using Printgraph CMOS I (ATTO, Japan) and visualized images were analyzed using Image J version 13.0.6 (NIH, USA).

### Statistical Analysis

Experimental data are expressed as the mean ± SD. Statistical differences were analyzed via a *t*-test using SPSS version 25.0 (IBM, USA). Values of *p* < 0.05 were considered significant.

## Results

### Effect of TJE on *S. aureus* Growth

**Fig. 1:**
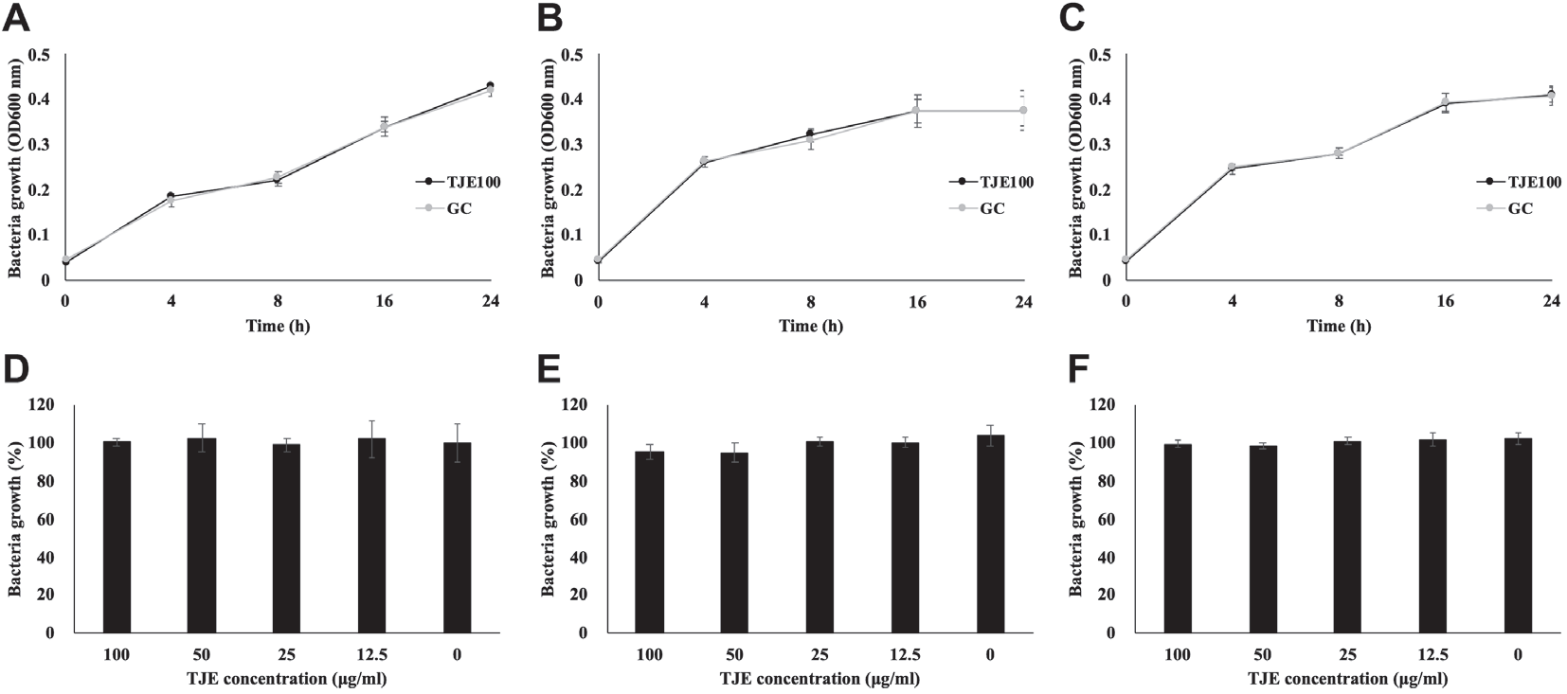
*Torilis japonica* ethanol extract (TJE) has no effect on the growth of MSSA KCTC 1927 (A, D), MRSA KCCM 40510 (B, E) and KCCM 40511 (C, F). *S. aureus* strains were pre-incubated for 18 h and then incubated with TJE for 24 h and OD_600_ was measured every 4 h. All experiments were performed in triplicates, and all values were expressed as the mean ± SD (*p < 0.05).

The effects of TJE on MSSA KCTC 1927, MRSA KCCM 40510, and MRSA KCCM 40511 was determined via bacteria viability assays and growth curve assay (Fig. 1). TJE did not affect the growth of all strains even at the highest concentration of 100 μg/ml. *T. japonica* extracts have been reported to show antibacterial activity against gram-positive and gram-negative bacteria such as *Bacillus subtilis* and *Klebsiella pneumoniae*, as well as antifungal activity against *Trichoderma* spp. [32, 33]. When the highest concentration (100 μg/ml) of TJE was used in this study, bacterial growth inhibition was not observed for the three strains; Stefanovic *et al*. [32], have reported that the MIC of TJE against *S. aureus* is 5 mg/ml. Therefore, the concentration of TJE used in this study was suitable for evaluating the inhibition of biofilm formation and QS regulation without affecting bacterial growth.

### Inhibition and Degradation of *S. aureus* Biofilm Production by TJE

**Fig. 2:**
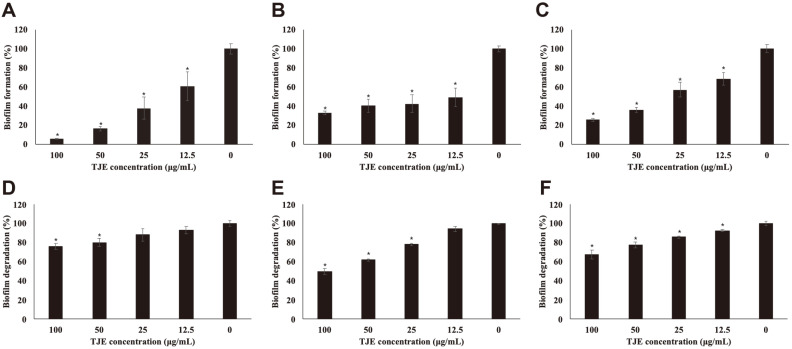
Effect of *Torilis japonica* ethanol extract (TJE) on MSSA KCTC 1927 (A, D), MRSA KCCM 40510 (B, E) and KCCM 40511 (C, F) biofilm formation and biofilm degradation. *S. aureus* strains were pre-incubated for 18 h and then incubated with TJE for biofilm inhibition. Formed biofilm was incubated with TJE for biofilm degradation. After incubation, Formed biofilm was measured by crystal violet dyeing method. All experiments were performed in triplicates, and all values were expressed as the mean ± SD (*p < 0.05).

Biofilm assays were performed to determine whether TJE could inhibit biofilm production or degradation in the *S. aureus* strains. The results of the biofilm inhibition and degradation assays of MSSA KCTC 1927, MRSA KCCM 40510, and MRSA KCCM 40511 treated with TJE are presented in Fig. 2. TJE significantly inhibited *S. aureus* biofilm formation (*p* < 0.05). The highest concentration (100 μg/ml) of TJE showed the highest inhibition of biofilm formation of 6.13% in MSSA KCTC 1927 (*p* < 0.05) and approximately 32% in MRSA KCCM 40510 and 25% in MRSA KCCM 40511 (*p* < 0.05). TJE also significantly inhibited biofilm formation of MSSA 1927, MRSA 40510, and MRSA 40511 when used at the lowest concentration of 12.5 μg/ml (*p* < 0.05). Mature biofilm was significantly degraded by TJE treated at more than 50 μg/ml (*p* < 0.05). *T. japonica* contains components such as quercetin, kaempferol and luteolin [27]. It has been reported that these components can inhibit the formation of *S. aureus* biofilm [40-42]. Therefore, the treatment of cultures with TJE inhibited biofilm formation and degraded *S. aureus* in a concentration-dependent manner; all TJE concentrations used in this assay significantly reduced biofilm formation compared to that of the control group (*p* < 0.05).

### Hemolysis Assay

**Fig. 3:**
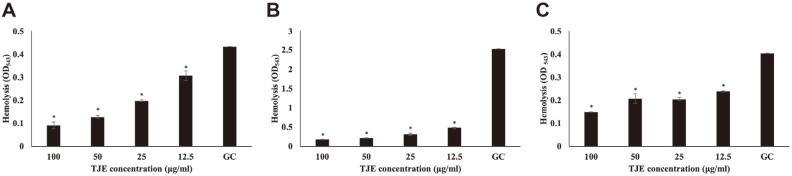
Effect of *Torilis japonica* ethanol extract (TJE) on sheep blood hemolysis by MSSA KCTC 1927 (**A**), MRSA KCCM 40510 (**B**) and KCCM 40511 (**C**). *S. aureus* strains were pre-incubated for 12 h and then incubated with TJE for 12 h. After incubation, culture supernatant and sheep blood were incubated for 1 h at 37°C. All experiments were performed in triplicates, and all values were expressed as the mean ± SD (*p < 0.05).

Based on the *agr* system, *S. aureus* is known to produce α-hemolysin and δ-hemolysin that cause hemolysis and affect biofilm formation [20, 21]. Therefore, hemolysis assays were performed to investigate the effects of TJE treatment on production of these proteins by the *S. aureus* strains. The results of hemolysis assays for MSSA KCTC 1927, MRSA KCCM 40510, and MRSA KCCM 40511 treated with TJE are presented in Fig. 3. The TJE was found to suppress hemolysis even at the lowest concentration of 12.5 μg/ml (*p* < 0.05), and hemolysis activity reduced via treatment with TJE in a concentration-dependent manner. Hemolysin is encoded by the *hla* gene encoded by the AgrA-*RNAIII* system [7, 20]. These results suggest that the TJE may reduce the production of virulence factors by downregulating AgrA-*RNAIII* system in the staphylococcal QS system.

### Effects of TJE on Staphylococcal Virulence Factor mRNA Expression

**Fig. 4:**
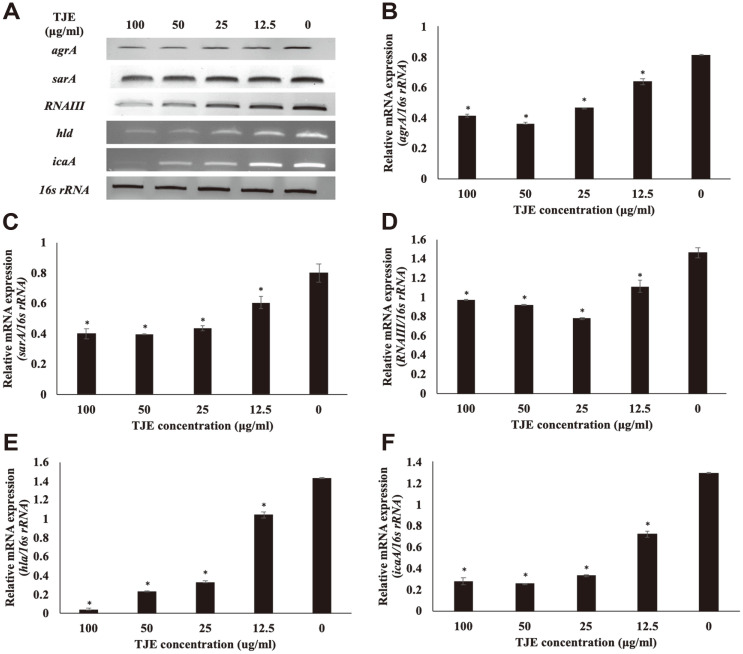
Effect of *Torilis japonica* ethanol extract (TJE) on the mRNA expression of virulence genes in MSSA KCTC 1927. *S. aureus* KCTC 1927 was pre-incubated for 12 h and then incubated with TJE for 12 h. All values are expressed as the mean ± SD. *p < 0.05 was significant difference from the relative mRNA expression of the non-treated control group.

**Fig. 6:**
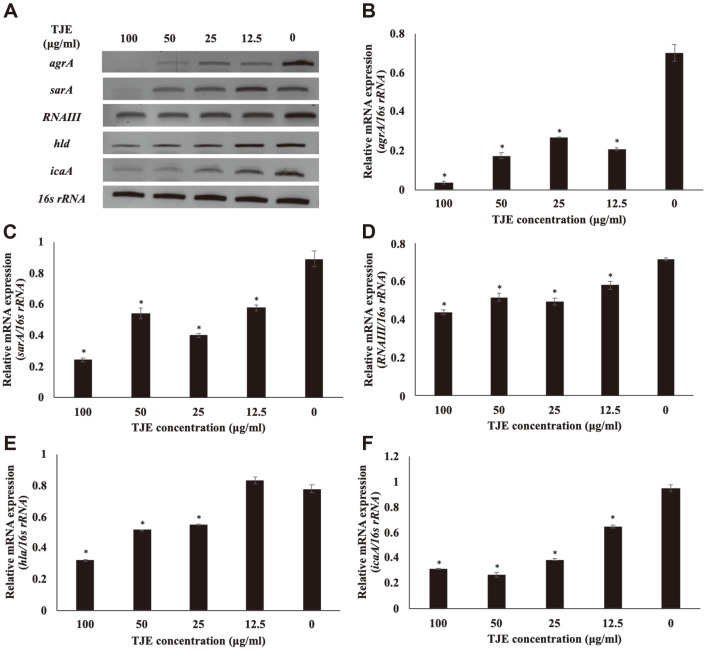
Effect of *Torilis japonica* ethanol extract (TJE) on the mRNA expression of virulence genes in MRSA KCCM 40511. *S. aureus* KCCM 40511 was pre-incubated for 12 h and then incubated with TJE for 12 h. All values are expressed as the mean ± SD. *p < 0.05 was significant difference from the relative mRNA expression of the non-treated control group.

After treating MSSA KCTC 1927, MRSA KCCM 40510, and MRSA KCCM 40511 with TJE, the mRNA expression of *agrA*, *sarA*, *icaA*, *RNAIII*, and *hla* was determined to investigate the effects of TJE on the *agr* system (Figs. 4 [Fig F5]–6). The TJE which inhibited biofilm formation was found to regulate the mRNA expression of *agrA*, *sarA*, *icaA*, *RNAIII*, and *hla* in *S. aureus*. The mRNA expression of *agrA* and *sarA* in TJE-treated *S. aureus* was significantly decreased compared to that in the control (*p* < 0.05). *RNAIII* was downregulated significantly in the same manner (*p* < 0.05). The expression level of *hla*, a gene associated with the production of hemolysin, was significantly decreased, which is in agreement with the result showing reduced hemolysis in *S. aureus* cultures treated with TJE (Fig. 3). In addition, *S. aureus* treated with TJE showed a significantly reduced mRNA expression of *icaA* (*p* < 0.05). The *icaA* gene is associated with intercellular adhesion ability. Down-expression of *icaA* gene is known to be related with inhibition of biofilm forming in *S.aureus* [22]. These results suggest that TJE may inhibit the biofilm formation and production of virulence factors in *S. aureus* by regulating the expression of *agrA*, *sarA*, *icaA*, *RNAIII*, and *hla* genes.

## Discussion

*S. aureus* is known to cause various diseases in humans and animals and is resistant to several types of antibiotics, which make these infections a public health concern [35-37]. MRSA strains are known to cause bacteremia that is difficult to treat. Continuous bacteremia is associated with a higher mortality rate than that of conventional bacteremia [38]. Due to the increase in incidence of infections caused by antibiotic-resistant bacteria, several recent studies have focused on biofilm inhibition and reduction of virulence via the use of new antibacterial substances derived from plant extracts [25]. *T. japonica* is a medicinal herb whose extracts have been reported to show synergistic effects with streptomycin or chloramphenicol against *S. aureus* [32]. In this study, after confirming that the TJE had no effect on the *S. aureus* growth, we performed biofilm and hemolysis assays. Moreover, this study demonstrated that *S. aureus* virulence and biofilm formation along with hemolysis may be suppressed by TJE treatment via downregulation of *agrA*, *sarA*, *RNAIII*, *hla*, and *icaA*. Recent strategies for preventing the development of antibiotic resistance have evaluated the regulation of virulence and biofilm formation, instead of using antibacterial agents. Because downregulation of *icaA* can interfere with QS and represents a new strategy for inhibiting biofilms [39], this study describes the inhibition of biofilms as well as the suppression of hemolysis and virulence using TJE, a natural product, and thereby suggests that the use of TJE may represent an effective strategy to overcome antibiotic resistance in *S. aureus*.

## Figures and Tables

**Fig. 5 F5:**
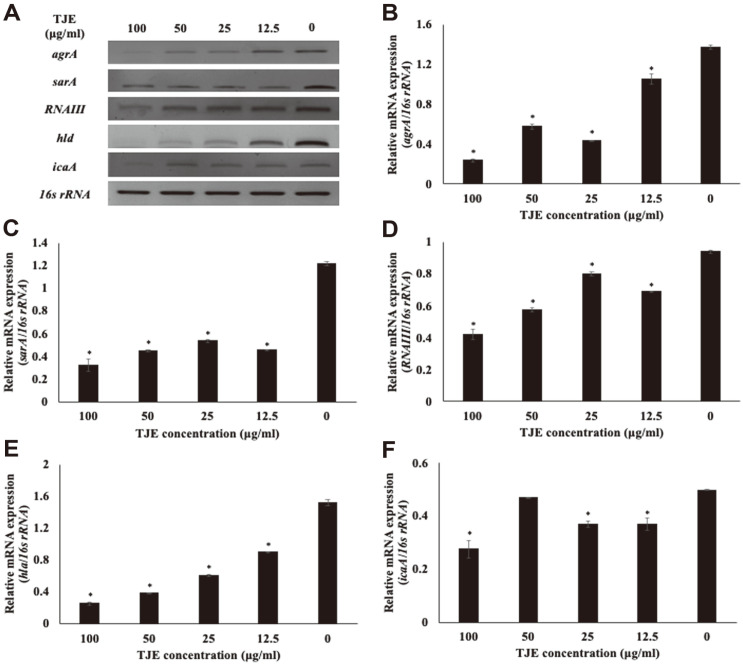
Effect of *Torilis japonica* ethanol extract (TJE) on the mRNA expression of virulence genes in MRSA KCCM 40510. *S. aureus* KCCM 40510 was pre-incubated for 12 h and then incubated with TJE for 12 h. All values are expressed as the mean ± SD. *p < 0.05 was significant difference from the relative mRNA expression of the non-treated control group.

**Table 1:** Primers used in polymerase chain reaction.

Gene	Size (bp)		Primer sequence (5′ to 3′)
*agrA*	554	F :	ATGAGATTAGGCATCGTTCC
		R :	TGGATGACAGTACCTGAGCC
*RNAIII*	235	F :	AAGCACCGTTACTATCTGCACA
		R :	GAGTAAATTTTGGTCGAATGCC
*sarA*	268	F :	CTGCAGAATGGGAAGTTATG
		R :	ACAAGTGAATTGAAACCGCC
*icaA*	270	F :	GCCATTGATGGTGATACGGTT
		R :	AGCCAAGCCTTGACGAACTAAAGC
*hla*	279	F :	GCCATTGATGGTGATACGGTT
		R :	AGCCAAGCCTTGACGAACTAAAGC
*16s rRNA*	318	F :	GCCATTGATGGTGATACGGTT
		R :	AGCCAAGCCTTGACGAACTAAAGC
